# What drives low‐income older adults' intention to use mobility applications?

**DOI:** 10.1111/ggi.14790

**Published:** 2024-01-02

**Authors:** Diana Yian Lian Chan, Chun Yong Chong, Pei‐Lee Teh, Shaun Wen Huey Lee

**Affiliations:** ^1^ School of Business Monash University Malaysia Bandar Sunway Malaysia; ^2^ School of Information Technology Monash University Malaysia Bandar Sunway Malaysia; ^3^ Gerontechnology Laboratory Monash University Malaysia Bandar Sunway Malaysia; ^4^ School of Pharmacy Monash University Malaysia Bandar Sunway Malaysia; ^5^ School of Pharmacy Taylor's University Lakeside Campus Subang Jaya Malaysia

**Keywords:** low‐income, mobility app, older adults, protection motivation theory, technology adoption

## Abstract

**Aim:**

Mobility applications have the potential to support low‐income older adults in facing mobility challenges. However, there is a generally lower uptake of technology in this segment. To understand factors affecting the intention to use a mobility app, we drew upon the Protection Motivation Theory, and tested a model of low‐income older adults' technology adoption.

**Methods:**

A cross‐sectional survey was conducted across seven states in Malaysia among community‐dwelling low‐income older adults aged ≥60 years old (*n* = 282). Measurement items were adapted from pre‐validated scales and 7‐point Likert Scales were used. Partial least squares structural equation modeling was utilized to assess the hypothesized model.

**Results:**

Mobility technology awareness was found to shape an individual's threat and coping appraisals associated with their intention to use a mobility app. The decision of a low‐income older adult to adopt a mobility app as a protective action is not a direct function of threat and coping appraisals but is indirect, and mediated by the underlying cost–benefit perceptions of non‐adoption and adoption of the mobility app. In terms of technology perceptions, perceived usefulness is a significant predictor, but not perceived ease of use.

**Conclusions:**

This study entails a new model by uncovering the psychological factors encompassing mobility technology awareness, threat‐coping appraisals, and cost–benefit perceptions on Technology Acceptance Model studies. These insights have important implications for the development and implementation of a mobility app among low‐income older adults. **Geriatr Gerontol Int 2024; 24: 342–350**.

## Graphical abstract

Low‐income older adults' intention to adopt a mobility app is not directly influenced by their threat and coping appraisals of facing mobility challenges but is indirect, mediated by the underlying cost–benefit perceptions of non‐adoption and adoption of the mobility app. Mobility technology awareness shapes an individual's threat and coping appraisals. Technology perception of usefulness is a significant predictor, but not ease of use.

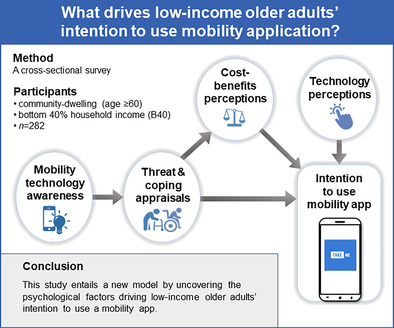

## Introduction

Mobility challenges have multifaceted implications, affecting older adults' ability to live independently and participate socially.^1^, ^2^, ^3^, ^4^, ^5^, ^6^ Low‐income older adults (LIOA) are more susceptible to mobility challenges.^7^, ^8^, ^9^, ^10^ In some cases, they have to rely on social services or community‐based care and assistance^11^ to live independently in their own homes. Technology‐mediated mobility solutions have the potential to promote healthy aging among LIOA. In this study, a new mobility app (MA), i.e., TakeMe app^12^, ^13^ integrates transport and volunteer services to provide door‐to‐door mobility services (i.e., assist wheelchair user; disability; carrying things) without any charges. This volunteer‐and‐community‐based technological intervention aims to support LIOA's mobility‐related needs including managing health (e.g., attending medical appointments, picking up prescriptions), access to essentials (e.g., grocery shopping), and facilitating social participation (e.g., attending social events). Nevertheless, studies found a generally lower uptake of technology, e.g., smartphone, gerontechnology, online health records among LIOA.^14^, ^15^, ^16^, ^17^ This issue is pertinent in technological interventions for aging in the low‐income context where successful implementation will contribute towards attaining healthy aging for all^18^ in line with the Sustainable Development Goals of leaving no one behind, particularly the most vulnerable groups.

There is a growing body of academic literature aimed at explaining LIOA's technology use behavior. Several studies^19^, ^20^, ^21^, ^22^ used theories (e.g., Technology Acceptance Model,^23^ Person‐Environmental Interaction Model,^24^ and Diffusion of Innovation Theory)^25^ whereas some studies focused on sociodemographic and environment factors.^19^, ^20^, ^26^, ^27^, ^28^ However, few studies examined perceptions (psychological factors) quantitatively.^20^, ^21^, ^29^ Most findings on perceptions were inductively derived qualitatively.^30^, ^31^, ^32^, ^33^ Thus, little is understood about the effect of LIOA's perceptions in shaping their intention to use technology. To the best of our knowledge, none of these studies investigated the technology context of an MA. More insights on how these factors interrelate could offer leverage points for policy makers, technology developers, and managers in the design and promotion of MAs among this segment.

The existing models explaining technology adoption among older adults in general, e.g., Senior Technology Acceptance Model,^15^ and the Model for the Adoption of Technology by Older Adults^34^ may be insufficient to encapsulate the LIOA and MA *user* and *usage* context because of two main reasons. First, the socioeconomic conditions are different.^35^ Owing to limited disposable income, there are likely different prioritizations and needs concerning technology use.^36^ Second, facing mobility challenges could mean a different set of consequences for LIOA when compared with their more affluent peers who potentially reach out to alternative resources, e.g., hiring a caregiver to assist in mobility needs and paying for ride‐hailing services.^37^, ^38^ Besides mobility‐related daily living needs, for a LIOA, mobility is vital for maintaining a sense of self and experiencing social capital, “It makes your life worthwhile. It gives you a purpose in living.”^39^ Therefore, in our *user* and *usage* context, we assert that adopting a utilitarian MA entails a protective behavior to mitigate the consequences of facing mobility challenges. Support for this assertion comes from technology adoption studies, e.g., healthcare wearables^40^; Mobile Chronic Disease Management Service^41^; and mHealth service^42^ wherein older adults' intention to use technology is a protection motivation. Therefore, we drew upon the Protection Motivation Theory^43^, ^44^ to explicate the intention to use MAs through a protective behavior perspective: when confronted with a threat, people assess the seriousness and susceptibility to the threat and evaluate the efficacies of coping response (i.e., threat and coping appraisals), which lead to behavioral intention to perform the recommended adaptive response.^45^, ^46^ Threat and coping appraisals are shaped by *intrapersonal sources of information* (e.g., experience).^45^, ^46^ Given older adults are generally less aware of new technologies that could be helpful to them,^47^ in our case of a new MA, some older adults lack an awareness of mobile ride‐hailing services and technology as potential solution to support mobility.^6^ Therefore, we will test mobility technology awareness as the antecedent to threat and coping appraisals shaping intention to use an MA.

A recent study on smartphone adoption urged any adoption study at the bottom‐of‐pyramid segment must not ignore the construct related to the monetary aspect.^36^ For decades, studies found that costs impede but benefits encourage technology use in low‐income aging contexts.^2^, ^27^, ^30^, ^33^, ^48^ These empirical findings point to cost–benefit perceptions as a salient determinant in LIOA's technology acceptance. Thus, this paper examines the role and effects of LIOA's cost–benefit perceptions to elucidate further the mechanisms underlying the intention to use MAs. Moreover, because older adults, digital immigrants, generally did not have life‐long exposure to technology in their formative years,^49^, ^50^ and digital skills vary by socioeconomic status,^51^ it is likely that LIOA may not find technology “useful” and “easy to use.” Hence, Technology Acceptance Model^23^ was employed to examine user's technology perceptions influencing intention to use MAs.

In summary, this study aims to examine a theoretical model (Figure S1) of mobility technology awareness, threat‐coping appraisals, cost–benefit perceptions, and technology perceptions in shaping the intention to use MAs among LIOA. The hypotheses of this study (Table S1) consist of three main processes. First, the intrapersonal source of information: Mobility technology awareness as the antecedent to threat‐coping appraisals. Second, the cognitive mediating process: threat‐coping appraisals and cost–benefit perceptions are drivers of intention to use MAs. Cost–benefit perceptions mediate the associations between threat and coping appraisals and intention to use MAs. Third, the coping mode: technology perceptions shape intention to use MAs.

## Methods

### Study design and participants

A cross‐sectional survey was conducted from May to August 2022. Purposive sampling was used for recruitment based on inclusion criteria: (i) community‐dwelling LIOA (age ≥60); (ii) bottom 40% household income classification (B40 segment)^52^ consistent with Malaysia low‐income literature^53^, ^54^, ^55^; (iii) able to answer questions in Malay, Chinese, or English; and (iv) voluntary participation. Participants were recruited from multiple sources: the Department of Social Welfare, older adult's activity centers (PAWE), and NGOs, across 10 urban/rural locations in seven states of Malaysia. We estimated we required a minimum sample of 114 participants (G*power version 3.1.9.7^56^, ^57^).

### Measures and procedures

The research model consists of 10 constructs (Table 1). To fit the mobility challenges and MA context, measurement items were adapted using pre‐validated scales with slight modifications^65^: mobility technology awareness,^58^ perceived severity,^66^, ^67^, ^68^ perceived vulnerability,^66^, ^67^, ^68^ self‐efficacy,^69^ response costs,^61^, ^66^ perceived costs of non‐adoption,^61^ perceived benefits of adoption,^70^, ^71^ perceived usefulness,^23^, ^72^ perceived ease of use,^73^, ^74^ and intention to use^75^ (Table S2). Seven‐point Likert Scales from “strongly disagree” to “strongly agree” were used to obtain the score of the items of each construct. A vignette briefly describing mobility challenges was adapted from previous study.^66^ The questionnaire was translated and back‐translated from English into Malay and Chinese language.^76^ The survey consists of two sections: (i) socio‐demographics, and (ii) measurement items. Participants experienced TakeMe app through video‐viewing and explored the prototype before evaluating the app. Surveys were researcher‐administered to eliminate literacy issues. Consent was obtained from all participants and confidentiality of data was guaranteed. This study was approved by the University Human Research Ethics Committee (Review Reference: 2022‐23 698‐78450).

**Table 1:** Main constructs in the research model

Process	Construct	Definition	Reference source
Intrapersonal source of information	Mobility technology awareness	The degree to which one is conscious about and interested in the availability of mobility technology (i.e., a mobility application) and how it could assist individuals in coping with mobility challenges	58, 59, 60
Threat appraisals	Perceived severity	How serious the individual believes that the threat (i.e., mobility challenges) would be to the individual's life	46
	Perceived vulnerability	How personally susceptible an individual feels to the communicated threat (i.e., mobility challenges)	46
Coping appraisals	Self‐efficacy	The perceived ability of the person to actually carry out the adaptive response (i.e., the ability to use mobility app)	43, 45
	Response costs	Overall costs (e.g., monetary, time, effort) associated with taking the adaptive coping response (i.e., using mobility app)	45 (p. 411)
Cost–benefit perceptions	Perceived costs of non‐adoption	One's perception of the expected *unfavorable* consequences, both monetary and non‐monetary *loss* from not performing the adaptive coping response (i.e., using mobility app)	61, 62
	Perceived benefits of adoption	One's perception of the expected *favorable* consequences, both monetary and non‐monetary *gains* from performing the adaptive coping response (i.e., using mobility app)	61, 62
Coping mode: user evaluations	Perceived usefulness	The degree to which a person believes that using a particular system would enhance his or her life	23
	Perceived ease of use	The degree to which a person believes that using a particular system would be free from effort	23
	Intention to use	Behavioral intention is a form of protection motivation to adopt the recommended behavior: the strength of one's intention to use mobility app	46, 63, 64

### Control variables

Demographic variables identified as factors influencing LIOA's technology adoption^19^, ^20^, ^26^, ^27^, ^28^ were included as control variables (Figure S1).

### Statistical analysis

Partial least squares structural equation modeling (PLS‐SEM) was employed to perform path modeling and testing of the hypothesized direct and indirect effects. Demographic data and the raw Likert Scale scores of each measurement items were imported into SmartPLS (v.4.0.8.5) to estimate the hypothesized relationships in the model. A two‐step process was conducted. First, a confirmatory composite analysis was performed to establish the reliability and validity of the construct measures.^77^, ^78^, ^79^, ^80^ Second, the SEM model was assessed for model fit using the standardized root mean square residual (<0.08),^77^, ^81^ size and significance of path coefficients,^77^ *f* ^2^ effect sizes,^56^ model's explanatory power using coefficient of determination (*R* ^2^) value,^82^ and model's predictive power (Q^2^_predict_ values) using PLS_predict_ procedure.^83^, ^84^ Bias‐corrected and accelerated (BCa) bootstrap method with 5000 bootstrap subsamples was applied for hypotheses testing. The BCa CI was used to detect mediation effects.^77^, ^85^

## Results

### Participant characteristics

This study yielded 282 (valid response rate: 95.9%) eligible survey responses. The mean age of participants was 68 ± 6 years. The majority were women (66.3%) and had at least primary education (90.1%). A summary of the participants' demographics are shown in Table 2.

**Table 2:** Descriptive statistics of sociodemographic and scores of the constructs (*n* = 282)

Variable		*n* (%) or mean ± SD
Gender	Male	95 (33.7)
	Female	187 (66.3)
Age, years	60–64	88 (31.2)
	65–69	78 (27.7)
	70–74	70 (24.8)
	75–79	30 (10.6)
	80–84	13 (4.6)
	≥85	3 (1.1)
Ethnicity	Malay^†^	165 (58.5)
	Chinese	54 (19.1)
	Indian	45 (16.0)
	Indigenous people	18 (6.4)
Household income	<RM2500	240 (85.1)
	RM2500–RM3169	24 (8.5)
	RM3170–RM3969	6 (2.1)
	RM3969–RM4849	12 (4.3)
Welfare beneficiary	Yes	135 (47.9)
	No	147 (52.1)
Education level	Never been to school	28 (9.9)
	Primary	110 (39.0)
	Secondary	105 (37.3)
	Diploma	23 (8.2)
	Tertiary	16 (5.7)
Assistive device use	Yes	47 (16.7)
	No	235 (83.3)
Self‐rated health	Excellent	14 (5.0)
	Very good	29 (10.3)
	Good	105 (37.2)
	Fair	106 (37.6)
	Poor	28 (9.9)
	Strata classification urban	112 (39.7)
	Rural	170 (60.3)
Living arrangement	Living alone	54 (19.1)
	Living with someone	228 (80.9)
Mobility technology awareness		5.41 ± 1.24
Perceived severity		6.49 ± 0.88
Perceived vulnerability		6.15 ± 1.13
Self‐efficacy		5.98 ± 0.68
Response costs		1.64 ± 0.90
Perceived costs of non‐adoption		6.32 ± 1.01
Perceived benefits of adoption		6.60 ± 0.54
Perceived usefulness		6.05 ± 0.47
Perceived ease of use		5.86 ± 0.71
Intention to use		6.38 ± 0.82

^†^ Malays are the largest ethnicity in Malaysia. 85.1% of participants came from households with monthly income <MYR2500 (near to the nation's poverty line income MYR2208.^51^

### Structural equation analysis of associations between mobility technology awareness, threat‐coping appraisals, cost–benefit perceptions, technology perceptions, and intention to use mobile apps

**Figure 1:**
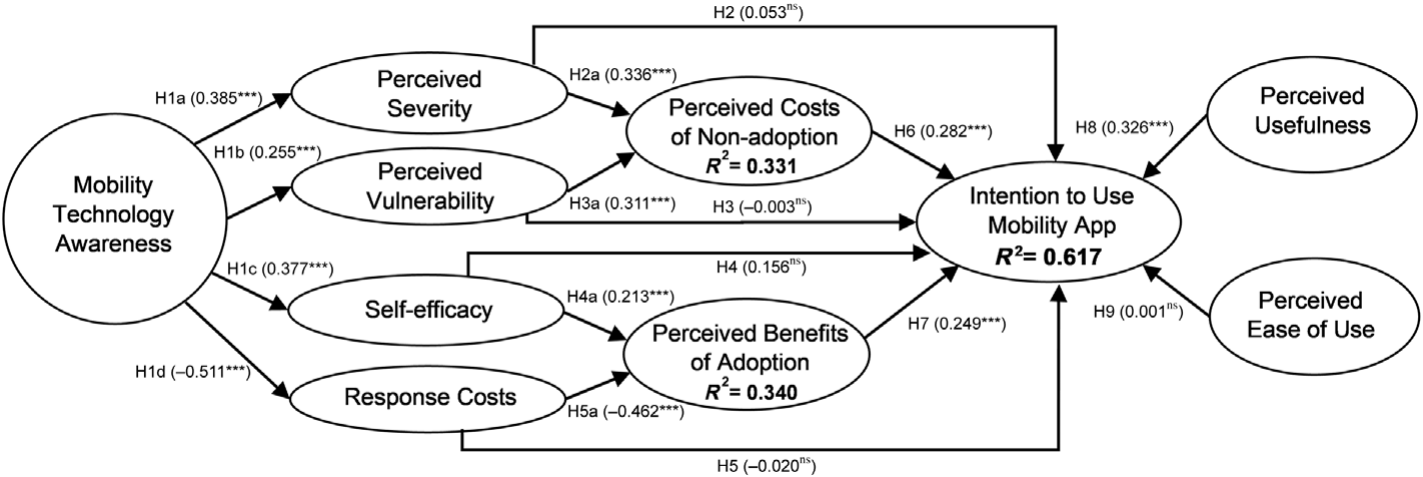
Factors affecting low‐income older adults' intention to use a mobility app. ***P < 0.001, **P < 0.01, *P < 0.05, ns, non‐significant; †specific indirect effect had marginally significant *P*‐value but 95% confidence intervals (0.013, 0.121) did not include 0^77^ (significant). The effects of demographic variables were gender (−0.064^ns^), age (−0.015^ns^), education (−0.034^ns^), household income (−0.008^ns^), health status (−0.029^ns^), assistive device use (−0.132^ns^), living arrangement (0.018^ns^), strata classification (−0.275***), and welfare beneficiary status (−0.203*).

The measurement models of the latent constructs demonstrate evidence of convergent validity, internal consistency reliability, and discriminant validity. The results of the structural model are depicted in Figure 1 and detailed in Tables 3 and 4. The hypothesized model depicted: (i) a reasonable model fit^77^, ^81^ (standardized root mean square residual = 0.069); (ii) explained a 61.7% variance of LIOA's intention to use MAs (*R* ^2^ = 0.617, near to substantial explanatory power)^82^ (Table S3); and (iii) a medium to high predictive power (Table S4).^83^, ^84^ Table 3 shows the results of the direct relationships. Mobility technology awareness had significant direct effects on all threat and coping appraisal constructs, supporting H1a–H1d. All the direct effects between threat‐coping appraisal constructs and intention to use were insignificant. Thus, H2–H5 were not supported. However, the associations between threat‐coping appraisal constructs and cost–benefit perceptions were significant, supporting H2a, H3a, H4a, and H5a. The direct effects of cost–benefit perceptions on behavioral intention were significant, supporting H6 and H7. In terms of technology perceptions, perceived usefulness significantly influences the intention to use the MA but the effect of perceived ease of use was insignificant. H8 was supported but not H9. Most of the exogenous variables demonstrate a medium to small *f* ^2^ effect sizes^56^ on the endogenous variables. The control variables were not significantly related to behavioral intention, except strata classification and welfare beneficiary status.

**Table 3:** Results of hypothesis testing of direct effects

Hypothesis	Path	β	Effect size *f* ^2^	95% CIs	Supported
H1a	Mobility technology awareness → perceived severity	0.385 (4.729)***	0.174§	[0.208, 0.534]	Yes
H1b	Mobility technology awareness → perceived vulnerability	0.255 (2.952)**	0.069‡	[0.090, 0.427]	Yes
H1c	Mobility technology awareness → self‐efficacy	0.377 (5.373)***	0.166§	[0.218, 0.497]	Yes
H1d	Mobility technology awareness → response costs	−0.511 (9.855)***	0.353¶	[−0.599, −0.391]	Yes
H2	Perceived severity → intention to use	0.053 (0.606) NS	0.004†	[−0.114, 0.235]	No
H3	Perceived vulnerability → intention to use	−0.003 (0.059) NS	0.000†	[−0.095, 0.104]	No
H4	Self‐efficacy → intention to use	0.156 (1.785) NS	0.034‡	[−0.003, 0.335]	No
H5	Response costs → intention to use	−0.020 (0.419) NS	0.001^n^	[−0.117, 0.072]	No
H2a	Perceived severity → perceived costs of non‐adoption	0.336 (4.030)***	0.111‡	[0.176, 0.500]	Yes
H3a	Perceived vulnerability → perceived costs of non‐adoption	0.311 (3.031)**	0.095‡	[0.113, 0.508]	Yes
H4a	Self‐efficacy → perceived benefits of adoption	0.213 (3.334)**	0.057‡	[0.079, 0.328]	Yes
H5a	Response costs → perceived benefits of adoption	−0.462 (7.904)***	0.270§	[−0.567, −0.338]	Yes
H6	Perceived costs of non‐adoption → intention to use	0.282 (4.046)***	0.106‡	[0.146, 0.414]	Yes
H7	Perceived benefits of adoption → intention to use	0.249 (2.981)**	0.081‡	[0.097, 0.419]	Yes
H8	Perceived usefulness → intention to use	0.326 (4.299)***	0.147‡	[0.172, 0.468]	Yes
H9	Perceived ease of use → intention to use	0.001 (0.009) NS	0.000†	[−0.139, 0.159]	No

*Note*: Significance level (bootstrapped): NS, non‐significant, ***P < 0.001, **P < 0.01, *P < 0.05, figures in parentheses are *t* values. *f* ^2^ effect size: †no effect, ‡>0.02 = small effect, §>0.15 = medium effect, ¶>0.35 = large effect. β = path coefficient; 95% confidence interval (95% CI) bias‐corrected through bootstrapping.

**Table 4:** Results of hypothesis testing: specific indirect effects (mediating effects)

Hypothesis	Path	β	95% CIs	Supported	Type of mediation
H6a	Perceived severity → perceived costs of non‐adoption → intention to use	0.095 (2.630)**	[0.041, 0.180]	Yes	Full mediation
H6b	Perceived vulnerability → perceived costs of non‐adoption → intention to use	0.088 (2.418)*	[0.029, 0.171]	Yes	Full mediation
H7a	Self‐efficacy → perceived benefits of adoption → Intention to use	0.053 (1.921)^†^	[0.013, 0.121]	Yes	Full mediation
H7b	Response costs → perceived benefits of adoption → intention to use	−0.115 (2.741)**	[−0.207, −0.045]	Yes	Full mediation

*Note*: Significance level (bootstrapped): ***P < 0.001, **P < 0.01, *P < 0.05, figures in parentheses are *t* values. β = path coefficient; 95% CIs = 95% confidence intervals bias‐corrected through bootstrapping. A full mediation is indicated in the case where the direct effect is non‐significant and the indirect effect is significant.^86^, ^87^, ^88^

^†^ Specific indirect effect had marginally significant *P*‐value (0.55) but 95% CIs (0.013, 0.121) did not include 0,^77^ thus we concluded that H7a was supported.

### Mediation of cost–benefit perceptions in the relationship between threat‐coping appraisals and intention to use mobile apps

Although the direct effects between threat‐coping appraisals and behavioral intention were insignificant, the indirect effects through cost–benefit perceptions were significant, supporting H6a, H6b, H7a, and H7b. Perceived costs of non‐adoption fully mediate the association between perceived severity→behavioral intention and perceived vulnerability→behavioral intention. Perceived benefits of adoption fully mediate the association between self‐efficacy→behavioral intention and response costs→behavioral intention (Table 4).

## Discussion

The purpose of this study is to examine the psychological factors (i.e., perceptions) driving LIOA's intention to use MAs. Our results supported the antecedent role of mobility technology awareness in shaping threat and coping appraisals, suggesting that the awareness of the availability of the MA attuned an individual to an acute sense of threat posed by consequences of mobility challenges, and elevated the perceptions about coping response. One practical approach to improve technology‐mediated mobility solutions adoption could be to increase the technology awareness among the population. For instance, short video clips can be created to increase the awareness of MAs in general (e.g., Grab,^89^ Jom Makcik,^90^ Teman Malaysia^91^) and how technological interventions can help support individuals facing mobility challenges.

In terms of threat appraisals, our results observed a deviation from past research, showing a negative association (despite insignificant) between perceived vulnerability and behavioral intention. The possible explanations are: older adults perceiving they are more vulnerable to the threat of mobility challenges may experience a reversal effect. Previous health protective behavior studies found higher levels of perceived susceptibility to threat can have variable effects, sometimes motivating preventive behavior but often leading to denial and avoidance.^92^, ^93^ A previous study found that Malaysian older adults generally avoided thinking or discussing about future care needs due to cultural taboos.^94^ Hence, perceived vulnerability may be less effective in predicting intention to use MAs in this context. Similarly, our results found insignificant positive associations between perceived severity and behavioral intention. This is an interesting discovery contrary to studies that showed older adults are generally aware of mobility challenges and that it is vital for independent living.^95^, ^96^ A possible reason for the non‐significant relationship is the research context in Malaysia, a collectivist rather than individualist, culture. In collectivist societies, individuals build strong relationships with other individuals within their family and within a kinship system.^68^, ^97^ The interdependence way of life^98^ − “*Bagai aur dengan tebing, adat hidup tolong menolong*” (Malay proverbs) meaning “Like the bamboo and the riverbank, the customs of the living are helping one another” − is an embedded culture. An earlier study also showed that among older adults living alone, instrumental assistance came from relatives, neighbors, friends, and having a system of looking out for each other.^94^ Besides, the Asian filial obligation has inculcated preferences and expectations for familial care.^94^, ^99^, ^100^ Given the cultural norms, although older adults perceived facing mobility challenges as severe, it might not engender the intention to use MAs, which relies on technology and volunteer assistance instead of receiving help from family or someone they knew. These reasons may explain why in this study, the perceived severity of facing mobility challenges did not directly instigate the intention to use MAs. This suggests that in the implementation and promotion of MAs among LIOA, solely communicating the noxiousness and susceptibility to the threat of facing mobility challenges is insufficient to motivate the uptake of MAs. Therefore, a paradigm shift in policies moving away from relying on familial care alone^100^ could be beneficial to induce change in the support for older adults, encouraging the acceptance of technological interventions as important alternatives.

While previous studies^45^, ^46^ indicated that coping appraisals are stronger predictors of behavioral intention, the present study showed that both self‐efficacy and response costs do not directly drive intention to use MAs. Similar to the threat appraisals, the findings might be attributable to our cultural context. For instance, the majority of our samples comprised of Malays who are Muslims, and they firmly adhere to Islamic spiritual practices.^101^ Past studies found that Islamic practices including, “*Alhamdulillah*,” which means contentment, and “*Redha*,” which means wholeheartedly accepting the decree of the Almighty were integral as their religious coping when facing challenges.^102^, ^103^ Therefore, even if they perceive a high self‐efficacy and lower costs of using MAs, the embedded religious coping^104^ of contentment and total acceptance might render coping through technological interventions as omittable.

The empirical results supported that cost–benefit perceptions have significant positive effects on intention to use MAs in terms of (i) a stronger intention when they perceive a passive act of *non‐adoption* would generate costs, losses, and disadvantages with the absence of a free mobility support when facing mobility challenges, and conversely, (ii) a higher intention if they believe that *adoption* is profitable, provide various benefits and gains in their limited‐resource situation. In particular, our results demonstrated that threat appraisals have significant associations with perceived costs of non‐adoption, whereas coping appraisals have significant associations with perceived benefits of adoption, indicating that threat‐coping appraisals are nonetheless important in forming cost–benefit perceptions. The results revealed that the decision of a LIOA to adopt MAs as a protective action is not a direct function of threat and coping appraisals but rather, is indirect and mediated by the underlying cost–benefit perceptions of not adopting or adopting the MAs. Notably, problems of living conditions, health and food insecurity persists among the low‐income B40 segment.^55^, ^105^, ^106^ Nearly half of our sample are welfare beneficiaries who strived to meet their basic needs for food and shelter.^94^ In such precarious context, the results revealed that cost–benefit perceptions were more salient in eliciting the uptake of a free MA in facing mobility challenges. Therefore, besides communicating the threat of facing mobility challenges as a stimulus for protection motivation, it is crucial for government agencies and service providers to emphasize on the potential benefits in economic terms in comparison with the expected costs and losses of not using MAs. In doing so, LIOA could have a clearer understanding of the possible gains and losses that motivate the uptake of technological interventions such as MAs.

Consistent with the previous LIOA study,^22^ perceived usefulness was found to be the most influential predictor of intention to use MAs whereas the effects of perceived ease of use was insignificant. Technology adoption studies focusing on LIOA also indicated a low level of perceived ease of use and usability issues as barriers of technology adoption.^22^, ^33^ A recent systematic review indicated that in limited resource contexts, LIOA are more likely to use technologies if there are necessities or fundamental needs that technologies can provide.^29^ Therefore, it is important to ensure the efficacies of technological design and interventions in addressing the needs of LIOA. Future development of mobile app‐based solutions for older adults should incorporate participatory design^13^ to yield design solutions that address their specific needs. Moreover, programs allowing older adults to test and experience the MA could be helpful for them to be acquainted with its usefulness and ease of use.

To the best of our knowledge, this is the first study examining the intention to use mobility apps among LIOA. Studies on technology adoption largely focus on older adults, and often neglect the low‐income segment. This study entails a new model by uncovering the psychological factors influencing intention to use MAs among LIOA. However, caution should be taken when applying our results to LIOA of different cultural context. Furthermore, the model was developed based on the usage context of an MA to address mobility‐related needs, which is pivotal for independent living in aging.^3^, ^5^ It would thus be useful for future research to test this model in different culture and technology usage context to verify the extent of its generalizability. Several limitations of this study should also be acknowledged. First, this study is cross‐sectional. Second, we did not capture the participants' previous experience in using smartphone or mobile app. The Hand Phone Users Survey 2021 report by the Malaysian Communications and Multimedia Commission shows smartphone adoption rate is >90% across all income categories,^107^ indicating a high smartphone penetration including among the low‐income. Nevertheless, the effect of experience or smartphone ownership should be controlled to ensure the robustness of the theoretical model in predicting behavioral intention. Furthermore, qualitative approaches would greatly enrich our understanding in terms of the *how* and *why* that underlies the relationships established in the model.

## Disclosure statement

The authors declare no conflict of interest.

## Supporting information


**Figure S1.** Research model of drivers of intention to use mobility app.
**Table S1.** Hypotheses.
**Table S2.** Measurement items.
**Table S3.** Assessing quality of the structural model by evaluating the explanatory power based on coefficient of determination (*R* ^2^), and predictive power (Q^2^_predict_).
**Table S4.** Q^2^_predict_ – Manifest variable prediction summary.

## Data Availability

All relevant data are presented in the paper and supplementary appendix.
