# Supplementary material for: What drives low‐income older adults' intention to use mobility applications?
Source: Geriatr Gerontol Int. 2024 Jan 2;24(Suppl 1):342–50. doi: 10.1111/ggi.14790 (PMC11503575; doi:10.1111/ggi.14790)
Supplement: Supplementary file 1 — Figure S1. Research model of drivers of intention to use mobility app. Table S1. Hypotheses. Table S2. Measurement items. Table S3. Assessing quality of the structural model by evaluating the explanatory power based on coefficient of determination (R 2), and predictive power (Q2 predict). Table S4. Q2 predict – Manifest variable prediction summary. [file GGI-24-342-s001.docx]

**Title: What Drives Low-Income Older Adults’ Intention to Use Mobility Application?**

**Supporting Information**

**Figure S1**

Research model of drivers of intention to use mobility app.

**
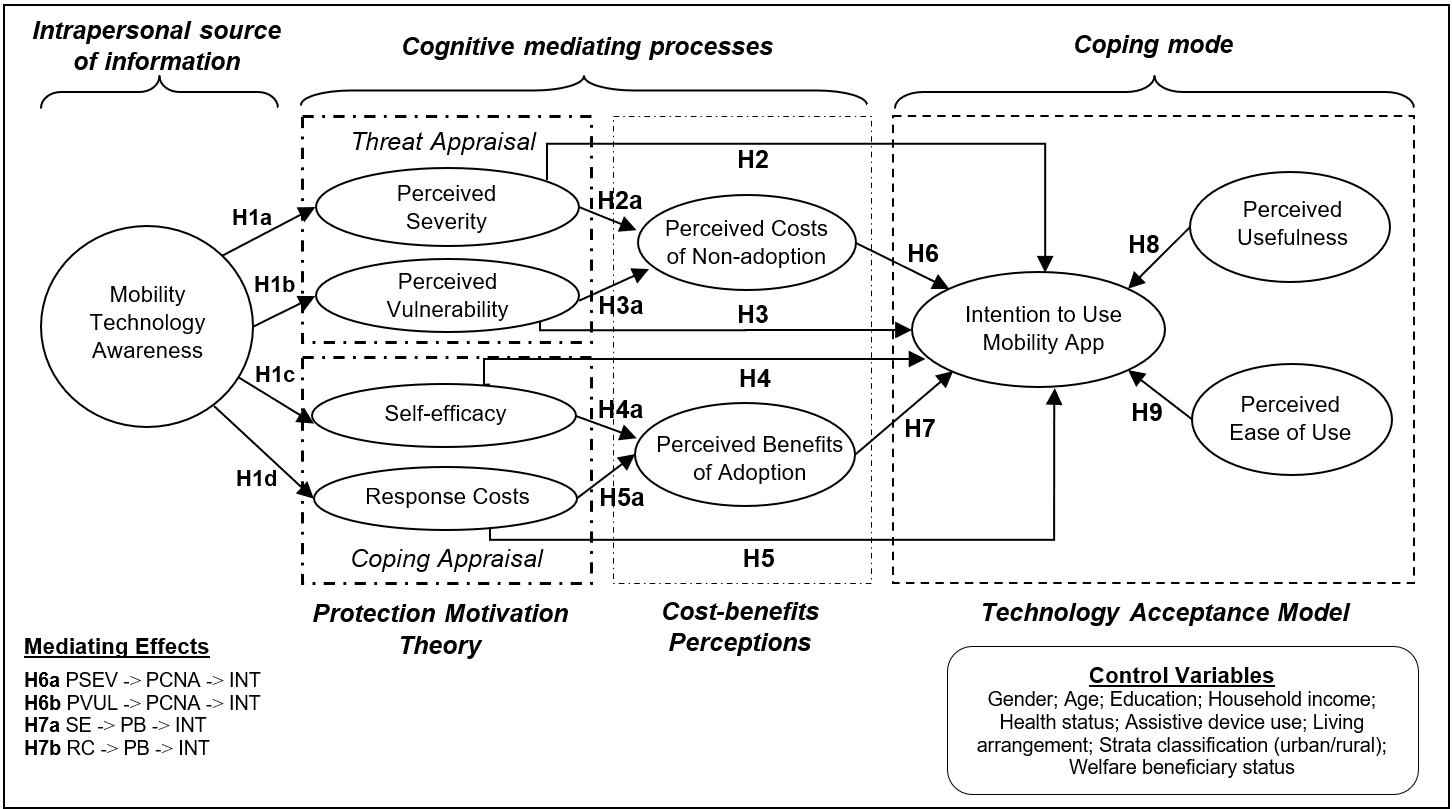
**

Abbreviations: PSEV = Perceived severity, PCNA = Perceived costs of non-adoption, INT = Intention to use, PVUL = Perceived vulnerability, SE = Self-efficacy, PB = Perceived benefits of adoption, RC = Response Costs

**Table S1** Hypotheses

| Processes | Hypothesis |  |
| --- | --- | --- |
| **Intrapersonal source of information:**  Mobility technology awareness as the antecedent to threat and coping appraisals | **H1a** | An older adult's mobility technology awareness is positively associated with perceived severity. |
|  | **H1b** | An older adult's mobility technology awareness is positively associated with perceived vulnerability. |
|  | **H1c** | An older adult's mobility technology awareness is positively associated with self-efficacy. |
|  | **H1d** | An older adult's mobility technology awareness is negatively associated with response costs. |
| **Cognitive mediating process:**  Threat and coping appraisals and cost-benefits perceptions are drivers of intention to use mobility app. Cost-benefits perceptions mediates the associations between threat and coping appraisals and intention to use mobility app | **H2** | An older adult’s perceived severity of mobility challenges is positively associated with intention to use mobility app. |
|  | **H3** | An older adult’s perceived vulnerability to mobility challenges is positively associated with intention to use mobility app |
|  | **H4** | An older adult's self-efficacy in using technology is positively associated with intention to use mobility app. |
|  | **H5** | An older adult’s response costs of adopting technology is negatively associated with intention to use mobility app |
|  | **H2a** | An older adult’s perceived severity of mobility challenges is positively associated with perceived costs of non-adoption. |
|  | **H3a** | An older adult’s perceived vulnerability to mobility challenges is positively associated with perceived costs of non-adoption. |
|  | **H4a** | An older adult’s self-efficacy in using technology is positively associated with perceived benefits of technology adoption. |
|  | **H5a** | An older adult’s response costs of adopting technology is negatively associated with perceived benefits of technology adoption |
|  | **H6** | An older adult’s perceived costs of non-adoption is positively associated with intention to use mobility app. |
|  | **H7** | An older adult’s perceived benefits of technology adoption is positively associated with intention to use mobility app. |
|  | **H6a** | Perceived costs of non-adoption mediates the positive association between perceived severity and intention to use mobility app. |
|  | **H6b** | Perceived costs of non-adoption mediates the positive association between perceived vulnerability and intention to use mobility app |
|  | **H7a** | Perceived benefits of adoption mediates the positive association between self-efficacy in using technology and intention to use mobility app. |
|  | **H7b** | Perceived benefits of adoption mediates the negative association between response costs of adopting and intention to use mobility app. |
| **Coping mode:** Technology perceptions shapes intention to use mobility app. | **H8** | An older adult's perceived usefulness of mobility app is positively associated with intention to use mobility app. |
|  | **H9** | An older adult's perceived ease of use of mobility app is positively associated with intention to use mobility app. |

**Table S2** Measurement items

| **Construct** | **Measurement Items** | **Sources** |
| --- | --- | --- |
| Mobility  Technology Awareness | MTA1: I follow news and development about mobility applications.  MTA2: I discuss with friends and people around me about benefits of using mobility applications.  MTA3: I read about the usefulness of mobility applications helping older adults’ mobility and transportation.  MTA4: I seek advice on mobility applications about how they can help older adults to live independently and participate socially.  MTA5: I am aware of the benefits of using mobility applications. | 57 |
| Perceived Severity | Please answer the following questions in terms of these mobility challenges: *(Vignette)* (1) driving or riding is no longer possible (2) family/ relatives/ friends/ neighbors could not provide transport for me (e.g., to buy groceries/ go for medical appointments) (3) physically, requiring assistance to move around  PSEV1: If I face mobility challenges, it would be severe.  PSEV2: If I face mobility challenges, it would be serious.  PSEV3: If I face mobility challenges, it would be significant. | 65,66,67 |
| Perceived Vulnerability | Please answer the following questions in terms of these mobility challenges: *(Vignette)* (1) driving or riding is no longer possible (2) family/ relatives/ friends/ neighbors could not provide transport for me (e.g., to buy groceries/ go for medical appointments) (3) physically, requiring assistance to move around  PV1: I am at risk of facing mobility challenges.  PV2: It is likely that I will face mobility challenges.  PV3: It is possible that I will face mobility challenges. | 65,66,67 |
| Self-efficacy | SE1: I could use mobility application to manage my mobility, if I had used a similar app before.  SE2: I could use mobility application to manage my mobility, if someone showed me how to.  SE3: I could use mobility application to manage my mobility, if I had time to try them out. | 68 |
| Response Costs | RC1: Using the mobility application is time consuming for me.  RC2: Using the mobility application is burdensome for me.  RC3: Using the mobility application is financially costly for me. | 60,67 |
| Perceived Costs of Non-adoption | Please answer the following questions in terms of these mobility challenges: *(Vignette)* (1) driving or riding is no longer possible (2) family/ relatives/ friends/ neighbors could not provide transport for me (e.g., to buy groceries/ go for medical appointments) (3) physically, requiring assistance to move around  PC1: My non-adoption of mobility application would create mobility impairment for me.  PC2: My non-adoption of mobility application would impact me negatively, such that I will be dependent on help from family/ friends.  PC3: My non-adoption of mobility application would create disadvantages for me, such that I would not have access to mobility assistance.  PC4: My non-adoption of mobility application would generate losses for me, such that I would have to bear the cost for using other transportation modes. | 60 |
| Perceived Benefits of Adoption | Please answer the following questions based on the door-to-door services of TakeMe app provided free by volunteers.  PB1: Using mobility application for my mobility and transportation will result in saving my personal time using transport services.  PB2: Using mobility application for my mobility and transportation will result in saving my personal expenses (i.e., money) using transport services.  PB3: Using mobility application for my mobility and transportation will result in convenience.  PB4: Using mobility application for my mobility and transportation will result in more independent living. | 69,70 |
| Perceived Usefulness | PU1: Using the TakeMe app will support my mobility in daily living.  PU2: Using the TakeMe app will help me to perform housekeeping (e.g., shop for groceries, prepare meals).  PU3: Using the TakeMe app makes it easier for me to manage my health (e.g., visit doctors, pick up prescriptions).  PU4: Using the TakeMe app makes it easier for me to maintain social connections (e.g., visit friends and family, attend religious services).  PU5: Overall, it is useful for me to use a mobility application. | 23,71 |
| Perceived Ease of Use | PEOU1: Using the TakeMe app is simple for me.  PEOU2: Learning to use the TakeMe app is easy for me.  PEOU3: The TakeMe app is easily understandable.  PEOU4: Overall, using the TakeMe app is convenient for me. | 72,73 |
| Intention to Use | IU1: If available, I intend to use the TakeMe app for mobility and transportation.  IU2: If available, I am likely to use the TakeMe app for mobility and transportation.  IU3: If available, I am certain that I would use the TakeMe app for mobility and transportation. | 74 |

Note: 7-point Likert Scales moving from “strongly disagree” to “strongly agree” were used.

**Table S3** Assessing quality of the structural model by evaluating the explanatory power based on coefficient of determination (R²), and predictive power (Q²_predict_)

| Main endogenous variables | R² | Q²_predict_ |
| --- | --- | --- |
| INT | 0.617 | 0.330 |
| PCNA | 0.331 | 0.066 |
| PB | 0.340 | 0.179 |

Note: INT = Intention to use, PCNA = Perceived costs of non-adoption, PB = Perceived benefits of adoption.

R² values of 0.67, 0.33, and 0.19 indicate substantial, moderate, and weak explanatory power, respectively.

All Q²_predict_ values were greater than the zero threshold.

**Table S4** Q²_predict_ - Manifest variable prediction summary

|  | Q²_predict_ | PLS-SEM_RMSE | PLS-SEM_MAE | LM_RMSE | LM_MAE |
| --- | --- | --- | --- | --- | --- |
| INT1 | **0.299** | 0.711 | 0.544 | 0.785 | 0.574 |
| INT2 | **0.198** | 0.785 | 0.578 | 0.792 | 0.601 |
| INT3 | **0.319** | 0.805 | 0.595 | 0.796 | 0.629 |
| PB1 | **0.115** | 0.701 | 0.494 | 0.75 | 0.522 |
| PB2 | **0.115** | 0.577 | 0.453 | 0.625 | 0.452 |
| PB3 | **0.148** | 0.523 | 0.432 | 0.552 | 0.429 |
| PB4 | **0.126** | 0.611 | 0.465 | 0.65 | 0.458 |
| PCNA1 | **0.054** | 1.242 | 0.817 | 1.388 | 0.914 |
| PCNA2 | **0.061** | 1.086 | 0.738 | 1.188 | 0.805 |
| PCNA3 | **0.052** | 1.056 | 0.728 | 1.094 | 0.763 |
| PCNA4 | **0.047** | 1.003 | 0.688 | 1.061 | 0.709 |
| PSEV1 | **0.087** | 0.932 | 0.614 | 1.083 | 0.671 |
| PSEV2 | **0.097** | 0.858 | 0.577 | 0.969 | 0.633 |
| PSEV3 | **0.131** | 0.868 | 0.592 | 1.037 | 0.651 |
| PVUL1 | **0.025** | 1.323 | 0.923 | 1.504 | 1.014 |
| PVUL2 | **0.037** | 1.228 | 0.854 | 1.365 | 0.927 |
| PVUL3 | **0.038** | 1.088 | 0.762 | 1.164 | 0.81 |
| RC1 | **0.196** | 1.107 | 0.762 | 1.159 | 0.795 |
| RC2 | **0.182** | 0.799 | 0.547 | 0.805 | 0.554 |
| RC3 | **0.113** | 1.124 | 0.816 | 1.161 | 0.822 |
| SE1 | **0.138** | 0.983 | 0.484 | 1.072 | 0.574 |
| SE2 | **0.032** | 0.712 | 0.394 | 0.715 | 0.368 |
| SE3 | **0.059** | 0.658 | 0.376 | 0.649 | 0.315 |
| Note: INT = Intention to use, MTA = Mobility technology awareness, PB = Perceived benefits of adoption, PCNA = Perceived costs of non-adoption, PEOU = Perceived ease of use, PSEV = Perceived severity, PU = Perceived usefulness, PVUL = Perceived vulnerability, RC = Response costs, SE = Self-efficacy.  PLS-SEM= partial least square structural equation modeling, RMSE = root mean squared errors, MAE = mean absolute error, LM = linear model. Lower RMSE (or MAE) values compared to the naïve LM benchmark for all indicators, majority indicators, minority indicators, or none of the indicators, indicates that the model has high, medium, low, or no predictive power, respectively. Results showed that the majority of indicators have lower RMSE (or the MAE) values compared to the naïve LM benchmark, thus this model has a medium to high predictive power. | | | | | |
